# Ectodermal-Neural Cortex 1 Down-Regulates Nrf2 at the Translational Level

**DOI:** 10.1371/journal.pone.0005492

**Published:** 2009-05-08

**Authors:** Xiao-Jun Wang, Donna D. Zhang

**Affiliations:** Department of Pharmacology and Toxicology, University of Arizona, Arizona, United States of America; University of Giessen Lung Center, Germany

## Abstract

The transcription factor Nrf2 is the master regulator of a cellular defense mechanism against environmental insults. The Nrf2-mediated antioxidant response is accomplished by the transcription of a battery of genes that encode phase II detoxifying enzymes, xenobiotic transporters, and antioxidants. Coordinated expression of these genes is critical in protecting cells from toxic and carcinogenic insults and in maintaining cellular redox homeostasis. Activation of the Nrf2 pathway is primarily controlled by Kelch-like ECH-associated protein 1 (Keap1), which is a molecular switch that turns on or off the Nrf2 signaling pathway according to intracellular redox conditions. Here we report our finding of a novel Nrf2 suppressor ectodermal-neural cortex 1 (ENC1), which is a BTB-Kelch protein and belongs to the same family as Keap1. Transient expression of ENC1 reduced steady-state levels of Nrf2 and its downstream gene expression. Although ENC1 interacted with Keap1 indirectly, the ENC1-mediated down-regulation of Nrf2 was independent of Keap1. The negative effect of ENC1 on Nrf2 was not due to a change in the stability of Nrf2 because neither proteasomal nor lysosomal inhibitors had any effects. Overexpression of ENC1 did not result in a change in the level of Nrf2 mRNA, rather, it caused a decrease in the rate of Nrf2 protein synthesis. These results demonstrate that ENC1 functions as a negative regulator of Nrf2 through suppressing Nrf2 protein translation, which adds another level of complexity in controlling the Nrf2 signaling pathway.

## Introduction

A variety of environmental toxicants, such as natural toxins, pollutants, heavy metals and chemical compounds, damage cells by generating reactive oxygen species (ROS) and thus disrupt the balance of cellular redox homeostasis [1], [2]. Loss of cellular redox homeostasis is regarded as oxidative stress, which elicits many cellular antioxidant responses [1]. One of the most important cellular antioxidant responses is regulated by Nrf2, a basic leucine zipper transcription factor. Nrf2 protects cells from environmental insults through transcriptional up-regulation of many antioxidant response element (ARE)-bearing genes including, antioxidant-encoding genes, phase II detoxifying genes, and phase III transporter genes [3], [4], [5], [6]. Coordinated induction of these genes allows cells to effectively neutralize and remove detrimental ROS to restore redox homeostasis and to avoid ROS-induced damage on cellular macromolecules, such as DNA, lipids, and proteins [5], [7], [8]. A growing body of evidence has associated ROS with the pathogenesis of many human diseases, such as cancer, neurodegenerative disease, cardiovascular disease, aging and inflammation [9], [10], [11], [12], [13]. Therefore, understanding how the Nrf2-dependent antioxidant response is controlled is of great significance in the development of novel strategies and chemopreventive drugs for the prevention and treatment of diseases [8], [12], [14], [15], [16].

In the past decade, much progress has been made in the understanding of the mechanisms by which the Nrf2 signaling pathway is efficiently regulated in response to oxidative stress or chemopreventive compounds such as sulforaphane (SF) [2], [17], [18]. The Neh2 domain, located at the amino terminus of Nrf2, is responsible for binding the critical negative regulator of Nrf2, Kelch-like ECH-associated protein 1 (Keap1) [2], [19], [20], [21]. Seven lysine residues in the Neh2 region have been shown to be essential for accepting Keap1-dependent ubiquitination by the Keap1-Cul3-Rbx1 E3 ubiquitin ligase complex, and is subsequently degraded by 26S proteasome [19]. Keap1, initially identified by a yeast two-hybrid system using Neh2 as a bait, is composed of five domains: N-terminal region, Broad complex, Tamtrack and Bric a brac domain (BTB), intervening region, Kelch, and C-terminal region [22]. The BTB domain has been demonstrated to be important for homodimerization of Keap1 and is also the region for Cul3 binding [19], [23]. The Kelch domain of Keap1 interacts with the Neh2 domain of Nrf2 [22]. Under normal conditions, low basal level of Nrf2 is achieved by constant degradation through the Keap1-Cul3-Rbx1 dependent ubiquitination and proteasomal degradation [2], [18], [24]. When cells are challenged by oxidative stress or treated with chemopreventive compounds, Keap1 acts as a stress sensor through its cysteine residues, in particular cysteine-151. Modification of certain cysteine residues leads to a conformational change of the E3 ubiquitin ligase, which significantly impairs and attenuates enzymatic activities of the E3 ligase. As a result, ubiquitination of Nrf2 and its degradation by the 26S proteasome decline rapidly. Nrf2 proteins translocate into the nucleus to switch on transcription of Nrf2-target genes, such as NAD(P)H quinone oxidoreductase (NQO1), glutathione *S*-transferase (GST), and heme oxygenase-1 (HO-1).

Regulation of gene expression is generally controlled at several levels: (i) control of gene transcription, (ii) control of post-transcriptional translation, (iii) control of protein abundance through ubiquitin-mediated protein degradation by the 26S proteasome, (iv) control of post-translational modifications of transcription factors, such as phosphorylation and acetylation, and (v) control of subcellular localization of transcription factors for its accessibility to target genes through cytoplasmic-nuclear trafficking. Among these regulatory levels, regulating Nrf2 abundance is a critical step, which can be achieved by transcription, translation, and protein stability. Keap1-mediated ubiquitination and proteasomal degradation of Nrf2 has been proven to be the primary control in turning on or off the Nrf2 signal. However, multiple regulatory mechanisms exist in controlling the activity of Nrf2. For example, phosphorylation of Nrf2 by different kinases was reported to play a role in controlling Nrf2 activities although mutagenesis studies have not identified the functional importance of any phosphorylatable residues [2], [25], [26], [27], [28]. Nucleocytoplasmic trafficking provides another level of regulation and is essential for turning off the Nrf2 signaling pathway following oxidative challenge [29]. Recently, our group has demonstrated that Nrf2 acetylation at multiple lysine residues located in the Neh1 DNA binding domain is important in fine-tuning the activity of Nrf2 [30]. Enhanced Nrf2 translation was observed when rat cardiomyocytes were treated with hydrogen peroxide although the detailed mechanism of how the Nrf2 translation increased remains unknown [31]. Enhanced transcription of Nrf2 was reported to contribute to an increase in Nrf2 protein when murine keratinocytic cells were challenged with anticarcinogen D3T [32]. The protein stability of Nrf2 is also controlled by the Neh6 domain of Nrf2 in an Keap1-independent mechanism in stressed cells [33]. A recent biochemical and genetic study on various Nrf2-inducing compounds using a zebra fish model demonstrates that many proteins may be involved in regulating the activity of Nrf2 [18]. Therefore, many proteins may work coordinately with Keap1 in controlling the activity of Nrf2 and its antioxidant responses.

BTB-Kelch proteins, containing BTB and Kelch domains at the amino and carboxyl termini respectively, make up a superfamily in mammals and invertebrates [34], [35]. Bioinformatics studies have identified over 41 genes in the human genome coding for BTB-Kelch proteins. While the function(s) of most BTB-Kelch proteins are unknown, some have been identified to function as substrate adaptors for the E3 ubiquitin ligase complexes that are required for ubiquitin-mediated protein degradation [36], [37], [38], [39]. Keap1 is the first BTB-Kelch protein identified to work as a substrate adaptor in the Cul3-based E3 ubiquitin ligase [19], [40], [41], [42]. Another BTB-Kelch protein KLHL12 was reported to target the D4 dopamine receptor to the Cul3-dependent E3 ubiquitin ligase for degradation, which is essential in controlling neurotransmitter dopamine signaling in the brain [39]. Previous studies have verified that ENC1 is able to form a functional complex with Cul3-Rbx proteins, resulting in auto-ubiquitination of ENC1. To clarify the function of BTB-Kelch proteins, we tested several BTB-Kelch proteins for their possible roles as substrate adaptor proteins to regulate Nrf2. Our results demonstrated that ENC1 markedly down-regulated the protein level of Nrf2 and the transcription of NQO1 and HO-1, but not the mRNA expression of Nrf2. Surprisingly, ubiquitination of Nrf2 was not altered in the presence of ENC1. Pulse-chase analysis showed that ENC1 reduced the rate of Nrf2 protein synthesis without affecting the stability of Nrf2. These results provide strong evidence indicating that ENC1 is a negative regulator of Nrf2.

## Materials and Methods

### Cell culture and transfection

MDA-MB-231 cells were purchased from ATCC and maintained in Eagle's minimal essential medium (MEM) supplemented with 10% fetal bovine serum (FBS). Cell cultures were incubated at 37°C in a humidified incubator containing 5% CO_2_. Transient cell transfection was carried out with Lipofectamine Plus (Invitrogen) according to the manufacturer's instructions.

### Construction of recombinant plasmid DNA

Construction of plasmids expressing wild-type ENC1-Myc, GAN1-Myc, sarcosin-Myc, HA-Nrf2, Keap1-CBD (chitin binding domain), HA-Ub (Ubiquitin) and Gal4-Neh2 proteins were previously described [43]. ENC1 deletion mutants, E320 and E570, were created by cloning PCR-generated DNA fragments in a pcDNA3.1 vector. The integrity of all cDNAs used in this study was confirmed by sequencing.

### Antibodies, immunoprecipitation and immunoblot analysis

Antibodies against Nrf2, Myc, HA, β-Actin, Tubulin, Gal4 (Santa Cruz Biotechnology), and CBD (New England Biolabs) were purchased from commercial sources. For detection of protein expression in total cell lysates, cells were washed with phosphate-buffered salt (PBS) buffer and lysed with sample buffer containing 50 mM Tris-HCl (pH6.8), 2% SDS, 10% glycerol, 100 mM DTT, 0.1% bromophenol blue at 48 hr post-transfection. Immunoprecipitation was performed with cell lysates in radio immunoprecipitation assay (RIPA) buffer (10 mM sodium phosphate, pH8.0, 150 mM NaCl, 1% Triton X-100, 1% sodium deoxycholate, 0.1% SDS) containing 1 mM DTT, 1 mM phenylmethylsulfonyl fluoride, and protease inhibitor mixture (Sigma). Cell lysates were incubated with 1 µg of antibodies and 10 µl of protein A-agarose beads at 4°C overnight. Immunoprecipitated complexes were washed four times with RIPA buffer and eluted in sample buffer by boiling for 5 minutes. Samples were resolved by SDS-polyacrylamide gels and transferred onto nitrocellulose membranes for immunoblot analysis.

### In vivo Ubiquitination assay

MDA-MB-231 cells were transfected with expression plasmids for HA-Ub and the indicated proteins. After 48 h, cells were lysed in a buffer containing 2% SDS, 150 mM NaCl, 10 mM Tris-HCl and 1 mM DTT. Cell lysates were boiled immediately for 10 minutes to inactivate cellular ubiquitin hydrolases and thus preserve ubiquitin-protein conjugates. The heated lysates were cooled down, diluted 5 times with a Tris-buffered salt (TBS) solution without SDS and used for immunoprecipitation with anti-Gal4 antibodies. Immunoprecipitated proteins were subjected to immunoblot analysis with antibodies against HA.

### Reporter gene activity assay

Plasmids for mouse GST-ARE-dependent firefly luciferase, control renilla luciferase (hRluc/TK from Promega), and other genes were co-transfected into MDA-MB-231 cells, followed by treatment with indicated chemicals for 16 hr. Cells were lysed at 48 hr posttransfection with passive lysis buffer (Promega) and the lysates were used to measure firefly and renilla luciferase activities using the Promega dual-luciferase reporter gene assay system.

### Real time RT-PCR

Total RNA extraction, first-strand cDNA synthesis and PCR conditions were reported previously [7]. Taqman probes were from the Roche universe probe library: hNrf2 (#70), hENC1 (#49), hKeap1 (#10), hNQO1 (#87), hHO-1 (#25), hGAPDH (#25). Oligo DNA primers were synthesized from IDT and the sequences are shown below.

hNrf2: forward acacggtccacagctcatc, reverse tgtcaatcaaatccatgtcctg;

hENC1: forward ccccacaatcaacaaatgg, reverse actactgcggcgttgctaa;

hKeap1: forward attggctgtgtggagttgc, reverse caggttgaagaactcctcttgc;

hNQO1: forward atgtatgacaaaggacccttcc, reverse tcccttgcagagagtacatgg;

hHO-1: forward aactttcagaagggccaggt, reverse ctgggctctccttgttgc;

hGAPDH: forward ctgacttcaacagcgacacc, reverse tgctgtagccaaattcgttgt.

All data were obtained from triplicate samples and presented as fold changes of mRNA levels, compared with the indicated controls in the graph.

### Pulse-chase analysis

Following incubation of MDA-MB-231 cells for 15 min in DMEM medium lacking methionine, cysteine and L-glutamine (Sigma), complemented with 10% dialyzed FBS, cells were cultured for 30 min in labeling medium containing 0.3 mCi/ml [^35^S]-methionine and [^35^S]-cysteine to label proteins. Cells were washed once with complete medium and cultured in complete medium at 37°C for the indicated chase time periods before cell lysis. Nrf2-containing pretein complexes were immunoprecipitated with anti-Nrf2 antibodies in RIPA buffer. Immunoprecipitated proteins were subjected to SDS-PAGE gel resolution and autoradiography.

## Results

### Nrf2 was down-regulated by ENC1

**Figure 1:**
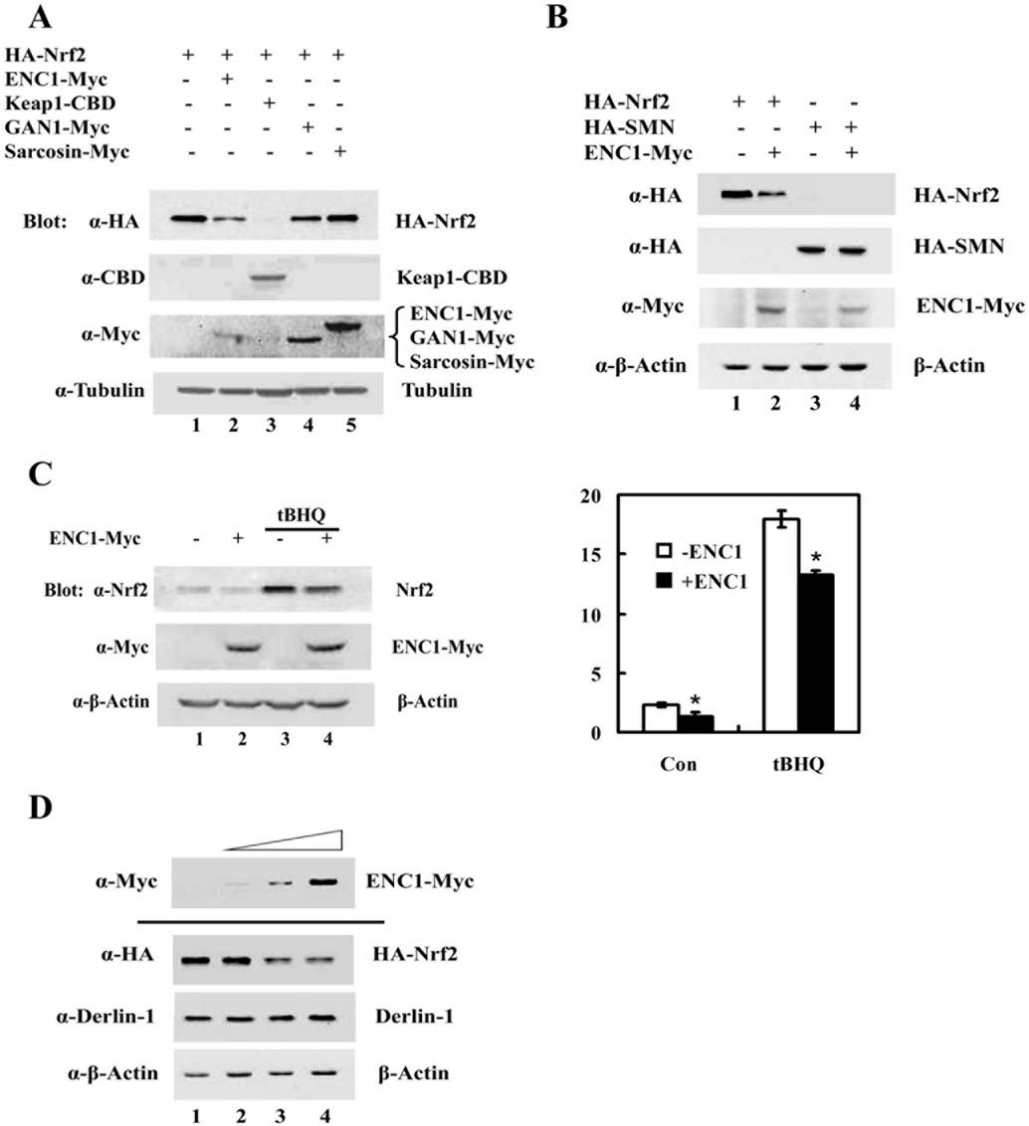
ENC1 down-regulated Nrf2. A. MDA-MB-231 cells were transfected with an expression vector for HA-Nrf2, along with an empty vector or a plasmid for ENC1-Myc, Keap1-CBD, GAN1-Myc or Sarcosin-Myc. Transfected cells were lysed at 48 hr post-transfection and cell lysates were subjected to immunoblot analysis with anti-HA, anti-Myc, anti-CBD and anti-tubulin antibodies. B. An expression vector for HA-Nrf2 or HA-SMN gene was transfected into MDA-MB-231 cells, along with an empty vector or an expression vector for ENC1-Myc. Cells were harvested and immunoblot analysis was performed. C. An expression vector for ENC1-Myc was transfected into MDA-MB-231 cells. Cells were left untreated or treated with 50 µM tBHQ for 16 hr before cell lysis. Cell lysates were subjected to immunoblot analysis (left panel). The intensities of the Nrf2 bands were quantified by the ChemiDoc XRS gel documentation system and Quantity One software from Bio-Rad (right panel). D. MDA-MB-231 cells were transfected with different amounts of an ENC1-Myc expression vector, along with an expression vector for HA-Nrf2. Cell lysates were subjected to immunoblot analysis with anti-HA and anti-Derlin-1 antibodies.

Bioinformatic analysis identified about 41 genes in the human genome that encode BTB-Kelch domain-containing proteins [34], [35]. It has been reported that each of the BTB-Kelch proteins, such as Keap1, ENC1, GAN1 or sarcosin, can function as a substrate adaptor by forming a complex with Cul3-Rbx 1 and is active in targeting self-ubiquitination of BTB-Kelch proteins [43]. To further investigate the possible effect of ENC1, GAN1 or sarcosin on Nrf2, MDA-MB-231 cells were transiently transfected with expression vectors for HA-Nrf2 and either ENC1-Myc, GAN1-Myc or sarcosin-Myc, and were subjected to immunoblot analysis. Keap1-CBD was included as a positive control. Interestingly, ENC1 and GAN1 reduced the protein level of Nrf2, although to a lesser degree, compared to Keap1 (Fig. 1A, compare lanes 2–4 with lane 1). In contrast, sarcosin had no effect (Fig. 1A, compare lane 5 with lane 1). In order to demonstrate that the ENC1-mediated down-regulation of Nrf2 is not due to an artifact from the expression vector, but rather specific in down-regulating Nrf2, a similar experiment was performed using HA-tagged survival motor neuron (HA-SMN) in place of HA-Nrf2. HA-SMN cDNA was cloned into the same cloning site using the same expression vector as HA-Nrf2. SMN is a gene correlated with loss of function of motor neurons of the spinal cord [44]. In contrast to HA-Nrf2, the level of HA-SMN was not reduced by overexpression of ENC1 (Fig. 1B, lane 3 and 4). Effects of ENC1 on endogenous Nrf2 were also examined in MDA-MB-231 cells transfected with ENC1-Myc alone. Nrf2 inducer *tert*-butylhydroquinone (tBHQ) was added to half of the samples for 4 hrs before cells were harvested to enhance Nrf2 levels. Cell lysates were subjected to immunoblot with anti-Nrf2 antibodies (Fig. 1C, left panel) and the Nrf2 band intensities were quantified and plotted (Fig. 1C, right panel). ENC1-mediated down-regulation of endogenous Nrf2 was observed in both untreated and tBHQ-treated samples (Fig. 1C, both left and right panels). To further confirm that ENC1 down-regulates Nrf2 specifically, cells were transfected with different amounts of an ENC1 expression vector, along with an expression vector for HA-Nrf2, and cell lysates were subjected to immunoblot analysis. ENC1 was able to down-regulate Nrf2 in a dose-dependent manner (Fig. 1D). Furthermore, overexpression of ENC1 did not elicit an unfolded protein response (UPR) as demonstrated by the same expression level of Derlin-1, an protein that is upregulated in response to UPR, among the samples (Fig. 1D). Collectively, these results demonstrated that ENC1 is able to down-regulate Nrf2 specifically.

### ENC1 down-regulated Nrf2 at the posttranscriptional level

**Figure 2:**
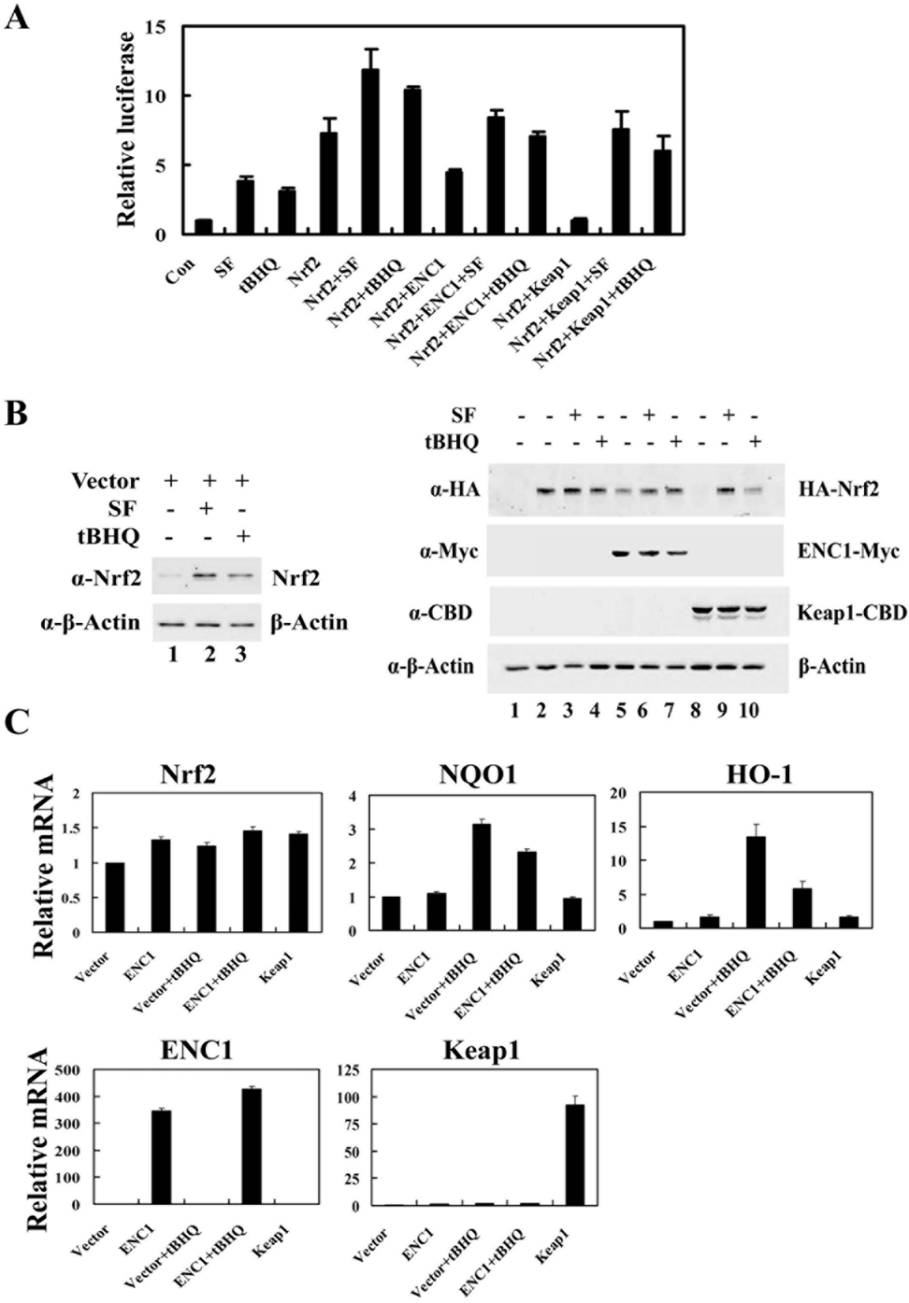
ENC1 down-regulated Nrf2 at the posttranscriptional level. A. MDA-MB-231 cells transfected with plasmids for ARE-driven firefly luciferase, TK-renilla luciferase and the indicated genes were treated with 7.5 µM SF or 50 µM tBHQ for 16 hr prior to cell lysis. Firefly and renilla luciferase activities were measured. Means and standard deviations were calculated from three independent experiments. B. Small aliquots of the lysates were subjected to immunoblot analysis with anti-Nrf2 (left panel), anti-HA, anti-Myc, anti-CBD and anti-β-actin antibodies (right panel). C. An empty plasmid or a plasmid for either ENC1-Myc or Keap1 was transfected into MDA-MB-231 cells and cells were treated with tBHQ for 16 hr. At 48 hr post-transfection, the relative mRNA levels of Nrf2, NQO1, HO-1, ENC1 and Keap1 were determined by real-time PCR.

Next, the effect of ENC1 on the transcriptional activity of Nrf2 was tested in the absence or presence of Nrf2 inducers. MDA-MB-231 cells were transfected with expression vectors for NQO1-ARE-luciferease reporter gene and HA-Nrf2, along with an expression vector for ENC1 or Keap1. Renilla luciferase reporter gene was used as an internal control. tBHQ or SF activated the transcriptional activity of endogenous Nrf2, demonstrating that the reporter gene system is functional (Fig. 2A). Similar to Keap1, ENC1 decreased transcriptional activities of Nrf2 in both untreated and treated conditions (Fig. 2A). Small aliquots of the same cell lysates were used for immunoblot analysis (Fig. 2B, both left and right panels). In agreement with the transcriptional activity of Nrf2, overexpression of ENC1 resulted in a decease in the Nrf2 protein level in both untreated and treated conditions (Fig. 2B, right panel, compare lanes 5–7 with lanes 2–4), indicating that ENC1 down-regulates the activity of Nrf2, primarily though reducing the protein level of Nrf2. However, the reduction of the transcriptional activity and the protein level of Nrf2 by ENC1 was not as substantial as that by Keap1 (Fig. 2A; Fig. 2B, right panel, compare lane 5 with lane 8), indicating that Keap1 is the primary regulator of the Nrf2-dependent cellular antioxidant response.

To explore the regulatory mechanism by which ENC1 down-regulates Nrf2, the mRNA levels of Nrf2 and two of its downstream target genes were determined using quantitative real-time RT-PCR in cells transfected with an expression vector for ENC1-Myc or Keap1 in the absence or presence of tBHQ. Similar to Keap1, ENC1 did not change Nrf2 mRNA expression in both treated and untreated conditions (Fig. 2C, Nrf2 panel). Under basal conditions, neither ENC1 nor Keap1 showed effects on mRNA expression of NQO1 and HO-1 (Fig. 2C, NQO1 and HO-1 panels), this is likely due to the low constitutive amount of Nrf2 and transcription of its downstream genes in this cell line. However, ectopic expression of ENC1 significantly decreased mRNA expression of NQO1 or HO-1 in cells treated with tBHQ (Fig. 2C, NQO1 and HO-1 panels, compare bar 4 with bar 3). Overexpression of ENC1 and Keap1 were confirmed by a robust induction of ENC1 and Keap1 mRNA (Fig. 2C, ENC1 and Keap1 panels). Taken together, these results demonstrate that ENC1 is able to suppress Nrf2 and its target gene expression without affecting Nrf2 mRNA levels. Thus, ENC1 down-regulates Nrf2 at the protein level, not at the transcriptional level.

### Down-regulation of Nrf2 by ENC1 was not through an ubiquitin-mediated proteasomal degradation mechanism

It has been well-established that Keap1 down-regulates Nrf2 through a ubiquitin-mediated proteasomal degradation machinery by functioning as a substrate adaptor for the Keap1-Cul3-Rbx1 E3 ubiquitin ligase. Suppression of Nrf2 by ENC1 is reminiscent of down-regulation of Nrf2 by Keap1. Since ENC1 has been shown to form a complex with Cul3-Rbx1 and may also function as a substrate adaptor, it is possible that Nrf2 may also be a substrate for the ENC1-Cul3-Rbx1 E3 ubiquitin ligase. Therefore the interaction between Nrf2 and ENC1 was determined by immunoprecipitation analysis with ectopically expressed HA-Nrf2 and ENC1-Myc. However, no interaction between these two proteins, even as overexpressed proteins, was detected (data not shown).

**Figure 3:**
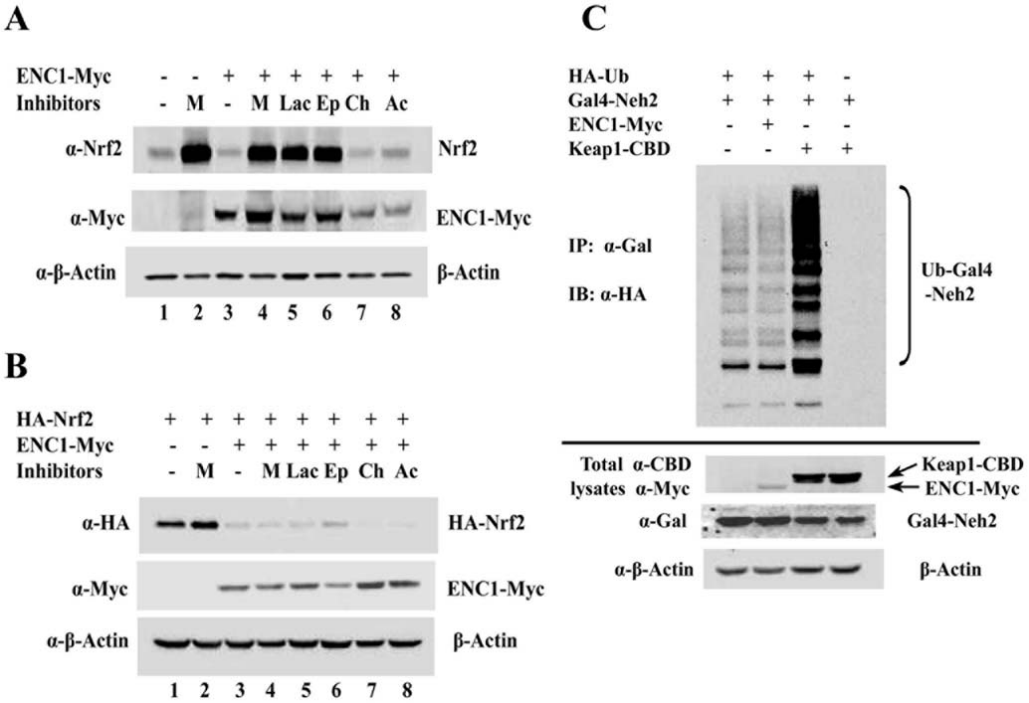
ENC1-mediated down-regulation of Nrf2 was independent of proteasomal or lysosomal degradation. A. MDA-MB-231 cells were transfected with an empty vector or a vector for ENC1-Myc. Transfected cells were treated with proteasome inhibitors MG132 (M) (10 µM), clasto-lactacystin β-lactone (Lac) (10 µM) or epoxomicin (Ep) (1 µM), or lysosome inhibitors chloroquine (Ch) (50 µM) or ammonium chloride (Ac) (50 mM) for 4 hr before cells were lysed at 48 hr post-transfection. Cell lysates were subjected to immunoblot analysis with anti-HA, anti-Myc and anti-β-actin antibodies. B. MDA-MB-231 cells were transfected with HA-Nrf2 and with an empty vector or ENC1-Myc. Transfected cells were treated with proteasome and lysosome inhibitors as described above. Immunoblot analysis was performed. C. *In vivo* ubiquitination assay was performed in MDA-MB-231 cells transfected with plasmids for HA-Ub and Gal4-Neh2, along with either ENC1-Myc or Keap1-CBD. Transfected cells were treated with 10 µM MG132 for 4 hr prior to cell lysis. Cell lysates were denatured by heating and subjected to immunoprecipitation with anti-Gal4 antibodies. The precipitated protein complexes were subjected to immunoblot analysis with anti-HA antibodies for detecting ubiquitin-conjugated Gal4-Neh2 (top panel). Small aliquots of total cell lysates were immunoblotted with the indicated antibodies (bottom three panels).

If the ENC1-mediated down-regulation of Nrf2 is through proteasomal degradation of Nrf2, then proteasomal inhibitors should be able to block the degradation of Nrf2 when ENC1 is overexpressed. Therefore, three proteasomal inhibitors, MG132, clasto-lactacystin β-lactone and epoxomicin, were tested for their ability to suppress the ENC1-induced decrease in Nrf2 expression. MG132 treatment resulted in a significant induction of endogenous Nrf2 in MDA-MB 231 cells (Fig. 3A, compare lane 1 with lane 2). However, the fold of induction by MG132 in mock-transfected MDA-MB 231 cells is equivalent to that in ENC1-transfected MDA-MB231 cells (Fig. 3A, compare lanes 3 and 4 with lanes 1 and 2). Similar levels of induction were observed when clasto-lactacystin β-lactone or epoxomicin was used (Fig. 3A, compare lanes 5 and 6 with lane 4). These results indicate that induction of Nrf2 in response to MG132 is due to blockage of Keap1-dependent proteasomal degradation of Nrf2 and ENC1-mediated down-regulation is unlikely through proteasomal degradation. Lysosome inhibitors, ammonium chloride and chloroquine, did not change Nrf2 levels (Fig. 3A, lanes 7, 8 and 3). These results clearly demonstrate that the ENC1-mediated down-regulation of Nrf2 is independent of proteasomal and lysosomal degradation machinery.

To further test this notion, a similar experiment was performed with overexpressed HA-Nrf2 to saturate endogenous Keap1, and therefore minimize the effect of Keap1 on Nrf2. MDA-MB-231 cells transfected with HA-Nrf2 and ENC1-Myc were treated with proteasomal and lysosomal inhibitors and the cell lysates were subjected to immunoblot analysis. Overexpression of ENC1 significantly reduced Nrf2 (Fig. 3B, compare lane 1 with lane 3). As expected, none of the inhibitors was able to restore the level of Nrf2 in ENC1-overexpressed cells (Fig. 3B, compare lane 4–8 with lane 3), indicating that the recovery of endogenous Nrf2 following proteasomal inhibition is solely due to blockage of the Keap1-mediated Nrf2 degradation (Fig. 3A, compare lanes 4–6 with lane 3). Taken together, these results show that neither the proteasome nor lysosome plays a role in the ENC1-mediated down-regulation of Nrf2.

Subsequently, a ubiquitination assay was performed to further prove that Nrf2 down-regulation by ENC1 is independent of the ubiquitin-mediated proteasomal degradation machinery. Overexpression of ENC1 did not increase ubiquitination of the transfected Neh2 domain of Nrf2 while Keap1 enhanced Neh2 ubiquitination significantly (Fig. 3C). This result shows that unlike Keap1, ENC1 does not participate in ubiquitination of Nrf2 and thus its down-regulation of Nrf2 is independent of ubiquitination and proteasomal degradation.

### Down-regulation of Nrf2 by ENC1 was independent of Keap1-ENC1 interaction

**Figure 4:**
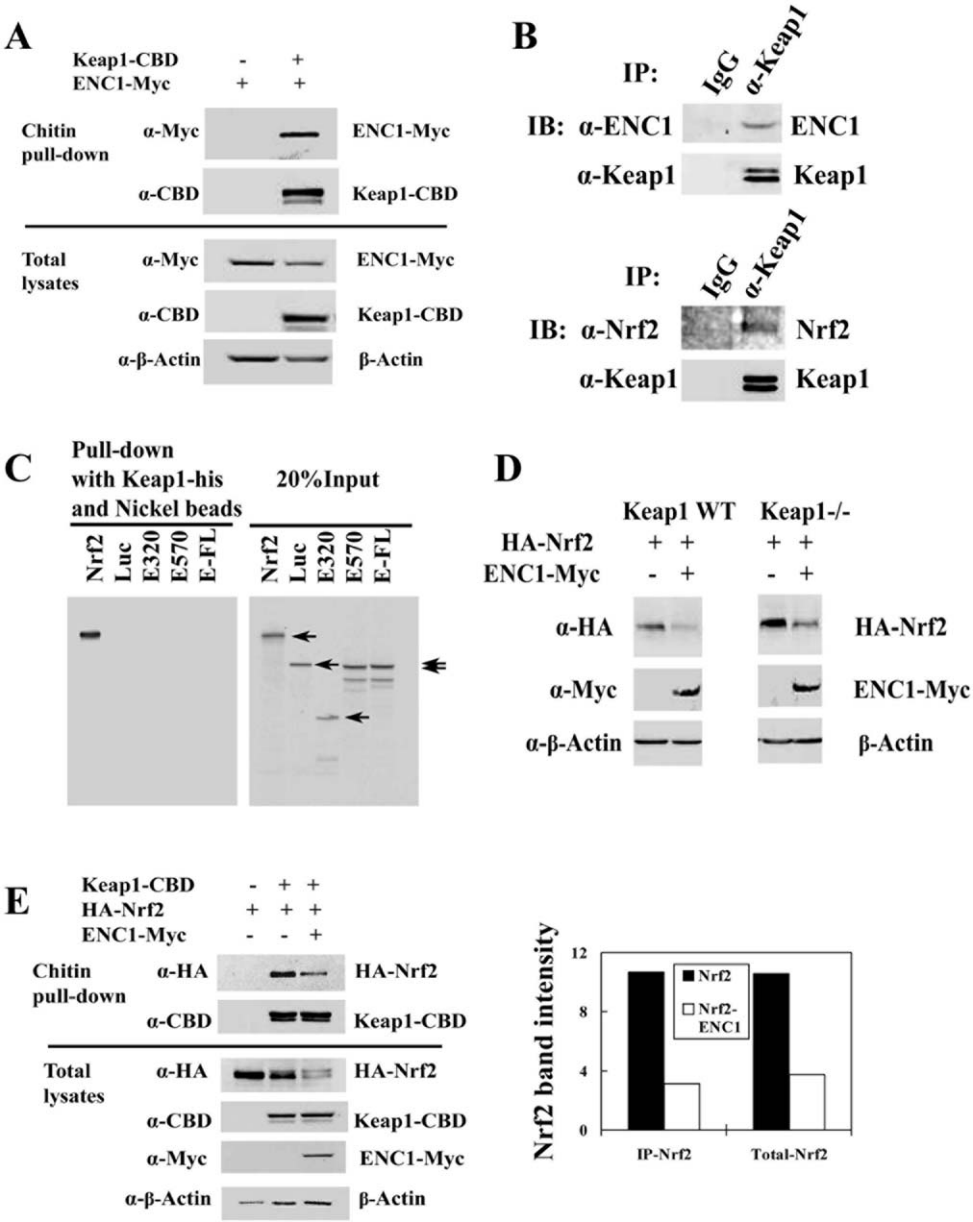
Keap1 was not essential in ENC1-mediated down-regulation of Nrf2. A. MDA-MB-231 cells were transfected with an expression vector for ENC1-Myc, along with an empty vector or an expression vector for Keap1-CBD. Cell lysates were subjected to chitin pull-down assay. Precipitated proteins were subjected to immunoblot analysis with anti-Myc and anti-CBD antibodies for detection of Keap1-CBD and ENC1-Myc (top panel). Small aliquots of total lysates were analyzed by immunoblot with the indicated antibodies (bottom panel). B. Cell lysates from MDA-MB-231 cells were immunoprecipitated with anti-Keap1 antibody. The precipitated protein complexes were subjected to immunoblot analysis with anti-Nrf2 and anti-ENC1 antibodies. IgG was included in the immunoprecipitation analysis as a negative control. C. Plasmids for full-length ENC1 (E-FL) and two ENC1 deletion mutants E320 and E570, Nrf2 and Luciferase (Luc) were used for *in vitro* transcription/translation to synthesize [^35^S]-labeled proteins. The proteins were incubated with Keap1-His purified from *E. Coli*. Protein complexes containing Keap1 were pulled-down with nickel beads and resolved in SDS-PAGE and detected by autoradiography. Nrf2 and Luciferase were used as a positive and a negative control. 20% of [^35^S]-labeled proteins were resolved by SDS-PAGE gel to show equivalent input of each protein (right panel). D. Keap1-/- and wild-type MEF cells were transfected with plasmids for HA-Nrf2 and ENC1-Myc. Cells were lysed at 48 hr post-transfection. Cell lysates were analyzed by immunoblot with anti-HA, anti-Myc and anti-β-actin. E. Cell lysates from MDA-MB-231 cells transfected with plasmids for Keap1-CBD, HA-Nrf2, and ENC1-Myc were used for pull-down assay with chitin beads Precipitated proteins were subjected to immunoblot analysis with anti-HA and anti-CBD antibodies (left figure, top two panels). Small aliquots of total lysates were analyzed by immunoblot with the indicated antibodies (left figure, bottom four panels). Nrf2 and β-Actin band intensities were quantified using Quantity One (BIO-RAD). The intensity of Nrf2 was normalized to that of β-actin (right graph).

To explore the mechanism of ENC1-mediated down-regulation of Nrf2, interaction of ENC1 with Keap1 was examined. Chitin pull-down assay was performed with transiently expressed Keap1-CBD and ENC1-Myc. Clearly, both Keap1-CDB and ENC1-Myc were present in CBD-bound complexes (Fig. 4A). Next, immunoprecipitation of endogenous Keap1 and ENC1 was performed with anti-Keap1 antibodies and Keap1 immunoprecipitates were subjected to immunoblot with anti-ENC antibodies. Immunoblot with anti-Nrf2 antibodies was also performed for this experiment as a positive control. Keap1 immunoprecipitates contained both Nrf2 and ENC1 (Fig. 4B, bottom and top panels). This encouraged us to test the direct interaction between ENC1 and Keap1. E.coli-expressed His-tagged Keap1 was incubated with ENC1 wild-type and two ENC1 deletion mutants that were synthesized *in vitro* in the presence of [^35^S]-methionine. Nickel beads were used to pull-down the Keap1-containing complexes. [^35^S]-labeled luciferase and Nrf2 were included as a negative and positive control. The results showed that ENC1 wild-type and the deletion mutants were not precipitated by Keap1-His (Fig. 4C), indicating that the association between Keap1 and ENC1 is indirect.

Since there was an indirect interaction between Keap1 and ENC1, the possible involvement of Keap1 in ENC1-mediated down-regulation of Nrf2 was examined. Keap1 wild-type and knockout mouse embryonic fibroblast (MEF) cells were transfected with HA-Nrf2 and ENC1-Myc to check if there is a difference in the ENC1-mediated Nrf2 down-regulation between wild-type and knockout cells. Overexpression of ENC1 was able to suppress the level of Nrf2 to a similar degree in both cell lines (Fig. 4D, left and right panels). This result suggests that Keap1 is not involved in ENC1-mediated down-regulation of Nrf2. The influence of ENC1 on the binding affinity between Keap1 and Nrf2 was also examined using chitin pull-down assay to characterize if ENC1 and Nrf2 compete with each other for Keap1 binding. Total Nrf2 level was reduced by Keap1 and was further decreased by ENC1 (Fig. 4E, left figure, lower panel, lanes 2 and 3). After pull-down with Keap1-CBD, the ratio of precipitated Nrf2 levels in the presence and absence of ENC1 (Fig. 4E, left figure, top panel, lanes 2 and 3) was comparable with that in total lysates (Fig. 4E, left figure, lower panel, lanes 2 and 3; right graphic). These results show that ENC1 does not influence the binding affinity of Nrf2 and Keap1 and that the ENC1-mediated down-regulation of Nrf2 is independent of the indirect interaction between Keap1 and ENC1.

### ENC1 down-regulated Nrf2 by decreasing the rate of Nrf2 protein synthesis

**Figure 5:**
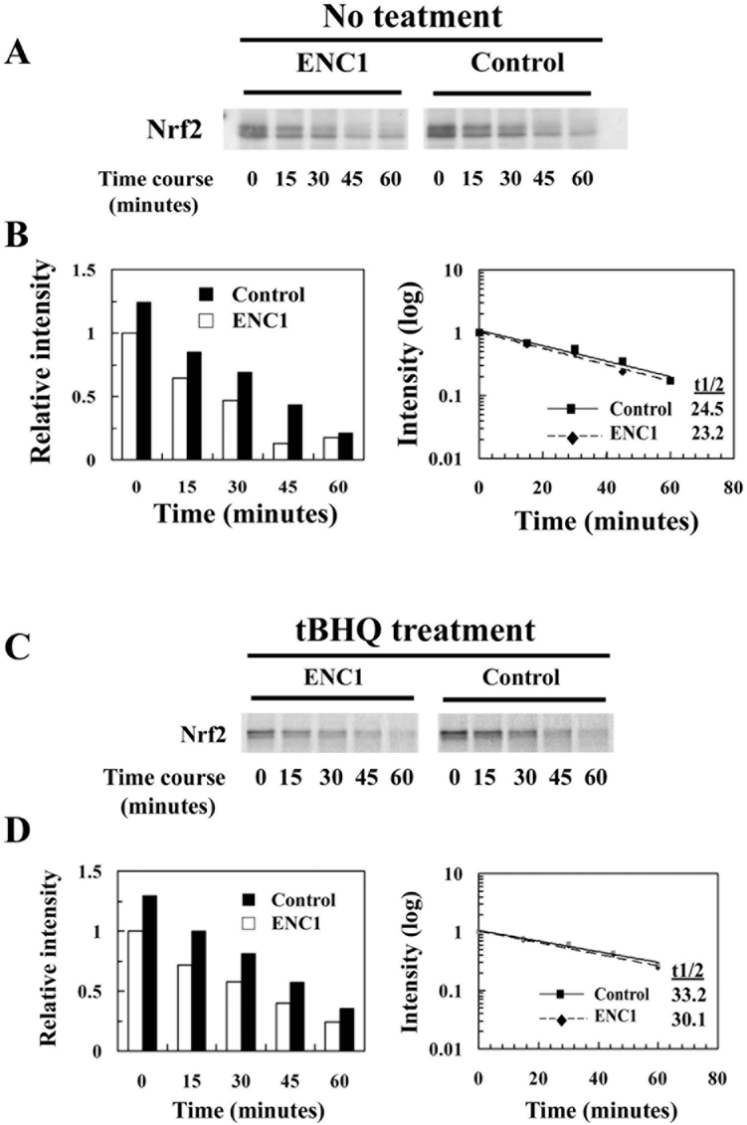
ENC1 down-regulated Nrf2 at the translational level. A. Pulse-chase assay was conducted with MDA-MB-231 cells transfected with either an empty vector or an expression vector for ENC1-Myc. Cells were incubated for 30 minutes with medium containing [^35^S]-methionine and [^35^S]-cysteine to label proteins. Cells were then washed and incubated in normal complete medium for the indicated time periods prior to cell lysis. Cell lysates were subjected to immunoprecipitation with anti-Nrf2 antibodies and immunoprecipitates were resolved in SDS-PAGE gel and detected by autoradiography. B. Nrf2 band intensities were quantified using Quantity One (BIO-RAD) and the half-life was plotted and calculated. C. Pulse-chase assay was conducted in the same way, except that cells were treated with 100 µM tBHQ for 4 hr. D. Quantified data of Fig. 5C.

The data show that reduction of Nrf2 by ENC1 was independent of proteasomal or lysosomal-dependent degradation. Furthermore, results from real-time PCR demonstrate that overexpression of ENC1 did not affect the mRNA level of Nrf2. Thus, it is most likely that Nrf2 modulation by ENC1 is at the level of protein synthesis. To test this, pulse-chase analysis was carried out in two sets of samples: vector-transfected or ENC1-Myc-transfected MDA-MB-231 cells were either left untreated or treated with tBHQ. Newly synthesized proteins were radio-labeled by incubating cells with medium containing [^35^S]-methionine and [^35^S]-cysteine for 30 minutes, and the cells were lysed at the indicated time points and subjected to immunoprecipitation using anti-Nrf2 antibodies. Immunoprecipitated Nrf2 proteins were resolved in SDS-PAGE gel. Results of autoradiographic analysis show that newly synthesized Nrf2 within a 30 minute-pulse time period was decreased in cells transfected with ENC1, compared to that in vector-transfected cells in both untreated and treated groups (Fig. 5A and 5C, compare first lanes, labeled 0 minute). Further, pulse-chase analysis also indicates that there was no change in Nrf2 half-life when ENC1 was overexpressed (Fig. 5B and 5D). Together, these results demonstrate that ENC1-mediated Nrf2 down-regulation is not through protein degradation, but rather through suppressing Nrf2 protein synthesis.

## Discussion

ENC1 and Keap1 both belong to the BTB-Kelch family and resemble each other in sequence and domain organization, indicating that ENC1 may have a similar role as Keap1 [43]. One of the major functions of Keap1 is as a substrate adaptor protein to target Nrf2 to the Cul3-based E3 ubiquitin ligase for ubiquitination and subsequent degradation by the 26S proteasome. Although few BTB-Kelch proteins have been demonstrated to function as a substrate adaptor for a E3 ubiquitin ligase, BTB-Kelch proteins have been demonstrated to have other functions not related to being substrate adaptors of E3 ubiquitin ligases. For example, the neuronal protein KRIP6, a BTB-Kelch protein, regulates the plasma membrane kainate receptor GluR6-mediated current in neurons through two different mechanisms [45]. One is modulating the association of GluR6 with the PDZ domain-containing protein, PICK1. Another mechanism relies on its direct binding with GluR6. Our study demonstrates that although ENC1 is able to down-regulate the protein level of Nrf2 and its downstream effects, its inhibitory function is independent of the ubiquitin-dependent proteasomal degradation pathway. The ENC1-mediated down-regulation of Nrf2 is accomplished through suppressing the rate of Nrf2 protein synthesis. Our results demonstrate that individual member of the BTB-Kelch family may have different functions and can regulate diverse cellular functions. Currently, how ENC1 is able to suppress the Nrf2 protein synthesis is unclear and further investigation is needed.

ENC1, also named NRP/B, is a nuclear matrix protein [46]. It is primarily expressed in the nervous system and has been demonstrated to be up-regulated during neuronal differentiation and is important for the differentiation process by interacting with active hypophosporylated p110 (RB) [46], [47]. In addition to regulating neuronal differentiation, ENC1 was also reported to be functionally important in regulating differentiation processes in adipocytes [48]. Disregulation of ENC1, such as mutation, overexpression, and mis-localization, was believed to contribute to brain tumorigenesis by enhancing cell proliferation, and inhibiting apoptosis [49], [50]. In tumor cells purified from patients suffering from hairy cell leukemia, ENC1 was significantly overexpressed in all the samples [51]. Interestingly, the cellular localization of ENC1 is controversial. Both nuclear and cytoplasmic localization has been reported. Nuclear localization of ENC1 is consistent with ENC1 being a nuclear matrix protein. However, ENC1 is also a member of BTB-Kelch family that interacts with actin through the Kelch domain and thus colocalizes with actin in the cytosol. We have detected cytoplasmic localization of four BTB-Kelch proteins, Keap1, ENC1, GAN-1, and sarcosin (data not shown).

Contradictory to our results demonstrating a negative effect of ENC1 on Nrf2 and its downstream genes, Avraham's group reported that ENC1 interacts with Nrf2 and upregulates NQO1 expression [52], [53]. The ENC1 cDNA used in our study encodes the exactly same amino acid sequence as the one used by Avraham's group. The discrepancy is unlikely due to the cell types used since same cell lines such as MDA-MB-231, COS and SH-SY5Y were used in the experiments (Our data with COS and SH-SY5Y cells were not shown). Ectopically expressed ENC1 wild-type was localized in the cytosol in our experimental settings, which is consistent with a report showing colocalization of ENC1 with actin filaments [48]. However, ectopically expressed ENC1 was detected exclusively in the nucleus in the studies from Avraham's group [52], [53]. In addition, we failed to detect interaction of ENC1 and Nrf2 in many cell types tested, although ENC1 interacts with Keap1 weakly and indirectly. It is most likely that the divergent results are due to the fact that we tagged Myc at the C-terminus of ENC1 while they used N-terminal Myc-tagged ENC1.
